# Genetic Polymorphisms of Angiotensin-Converting Enzyme 1 (ACE1) and ACE2 Associated With Severe Acute Respiratory Syndrome COVID-19 in the Palestinian Population

**DOI:** 10.7759/cureus.67670

**Published:** 2024-08-24

**Authors:** Lama AbuSaleh, Suheir Ereqat, Amer Al-Jawabreh, Abedelmajeed Nasereddin

**Affiliations:** 1 Biochemistry and Molecular Biology Department, Faculty of Medicine, Al-Quds University, Jerusalem, PSE; 2 Biochemistry and Molecular Biology Department, Al-Quds University, Jerusalem, PSE; 3 Medical Laboratory Sciences Department, Faculty of Allied Health Sciences, Arab American University, Jerusalem, PSE; 4 Infectious Disease Department, Al-Quds University, Al-Quds Bard College, Jerusalem, PSE

**Keywords:** covid-19, angiotensin-converting enzyme 2 (ace2), next-generation sequencing (ngs), severity of disease, genetic analysis, ace1, ras (renin angiotensin system)

## Abstract

As a key enzyme of the renin-angiotensin system (RAS), angiotensin-converting enzyme 2 (ACE2) is a validated receptor for SARS-CoV-2, linking RAS to COVID-19. Functional ACE1/ACE2 gene polymorphisms likely cause an imbalance in the ACE1/ACE2 ratio, triggering RAS imbalance and may contribute to COVID-19 complications. This study aimed to investigate four single nucleotide polymorphisms (SNPs) of ACE1 and ACE2 genes, three for ACE1 (rs4343, rs4342, rs4341) and one for ACE2 (rs2285666), in patients with COVID-19 among the Palestinian population. A total of 130 blood samples were collected, including 50 negative controls without COVID-19 infection, 50 cases with COVID-19 infection but not hospitalized, and 30 patients with severe COVID-19 infection hospitalized in the intensive care unit. Fragments of the ACE1 and ACE2 genes, including the targeted SNPs, were amplified using multiplex PCR and subsequently genotyped by next-generation sequencing with specific virtual probes. Our results revealed that ACE2 rs2285666 GG genotype carriers were more prevalent in COVID-19 patients compared to the control group (P=0.049), while no statistical differences were observed in the distribution of ACE1 (rs4343, rs4342, rs4341) variants between COVID-19 patients and the control group. GA carriers of ACE2, rs2285666, among cases and ICU groups were at lower risk of getting COVID-19 infection (P=0.002 and P=0.013, respectively), and they were unlikely to develop fatigue (P=0.043), headache (P=0.007), loss of smell (P=0.028), and dyspnea (P=0.005). Age and comorbidities such as hypertension and coronary artery disease (CAD) were independent risk factors for COVID-19 disease. Symptoms of COVID-19 patients such as fatigue, headaches, runny noses, and loss of smell were significantly higher in non-hospitalized cases of COVID-19, while dyspnea was more frequent in the ICU patients. In conclusion, these findings indicate that the ACE2 rs2285666 GG genotype is associated with an increased risk of COVID-19 infection. This association suggests a potential genetic predisposition linked to the ACE2 gene, which may influence the susceptibility and severity of the disease.

## Introduction

In late 2019, severe acute respiratory syndrome coronavirus 2 (SARS-CoV-2), the causative pathogen of coronavirus disease 2019 (COVID-19), spread widely throughout the world. The World Health Organization (WHO) declared COVID-19 as a global pandemic. On July 12, 2023, about 6.9 million people died from this highly contagious and pathogenic disease, while about 767 million cases have been confirmed worldwide [1].

SARS-CoV-2-enveloped virus is characterized by a positive single-stranded non-fragmented RNA genome containing 29.95-30.2 kb, and it is approximately 60-140 nm in diameter [2], with five genes ordered as 5'-replicase genes, spikes (S), envelopes (E), membranes (M), and nucleocapsids (N)-3’ [3]. SARS-CoV-2 possesses a transmembrane spike glycoprotein consisting of subunits S1 and S2 that are responsible for binding to the host cell receptor and facilitating the fusion of the virus with the host cell membrane. In order for SARS-CoV-2 to enter the body, spike protein must bind to angiotensin-converting enzyme (ACE2).

ACE2 is a multifunctional protein that has multiple roles in disease and health. It is a carboxy-aminopeptidase that converts angiotensin II (Ang II) into angiotensin (ANG 1-7) in the renin-angiotensin system (RAS) [4,5]. ACE2 is widely distributed across tissues, including the heart, the vascular system, the digestive tract, the lungs, the kidneys, and the nervous system. The widespread expression of this gene might explain the pathological manifestations and multi-organ system disease manifestations seen in patients with severe clinical outcomes [6]. As a result of SARS-CoV-2 binding to ACE2, the virus enters host cells through two routes: direct membrane fusion and endocytosis [7]. In spite of the fact that the membrane fusion pathway is 100-1000 times more potent than the endocytosis pathway (as measured for SARS-CoV) [8], protease expression determines how the virus gets into different cells [9]. Consequently, ACE2 binds to ectodomain S1 of SARS-CoV-2 in order to initiate COVID-19-induced inflammation. A decrease in ACE2 levels will happen after membrane fusion and the metabolism of angiotensin II (Ang II) will be disrupted [10]. When Ang II levels are elevated, inflammatory cytokines are released, and local inflammation occurs [11]. As a result of pulmonary vascular inflammation, ACE1 is shed more readily and released more into the interstitium, causing Ang II production and leukocyte infiltration to accelerate [12]. As a result of angiotensin II receptor type 1 (Ang II/ATR1) interaction overexpression, reactive oxygen species (ROS) production is increased, which aggravates systemic infection due to increased production of tumor necrosis factor-alpha (TNF-a), interleukin-6 (IL-6), and C-reactive protein (CRP) [13].

COVID-19 severity may be influenced by several single nucleotide polymorphisms (SNPs) located on ACE2 that affect its expression. Ten ACE2-related variants in coding, noncoding, and regulatory sites may explain the epidemiological differences associated with COVID-19. East Asian population had a higher prevalence of the variants associated with ACE2 upregulation (rs182366225 and rs2097723), whereas native populations from Amazon were found only to possess variants rs1027571965 and rs889263894 associated with ACE2 upregulation. The later population, however, was more likely to have "rs2285666" and "rs35803318". Three relevant polymorphisms (rs147311723, rs142017934, and rs4646140) were found to be more common in Africans, of which “rs142017934” was associated with gene upregulation and was exclusively found in this population. There is, however, an allele (rs5934250) that appears to lower ACE2 expression in some tissues in Europeans and some Africans. There are ACE2 variants "rs4240157, rs6632680, rs4830965, rs1476524, and rs2048683" that have been linked with higher tissue-specific expression of ACE2, leading to hospitalization, while the "rs1548474" variant was associated with low tissue expression and fewer complications [14-16].

However, rs2285666 has been tested clinically [17,18]. G8790A (rs2285666), one of the functional SNPs identified on ACE2, is located on chromosome X,p22, intron three and is likely to affect ACE2 gene expression by altering mRNA splicing [19]. It may be postulated that COVID-19 is more prevalent among carriers of specific genotypes of the A2350G (rs4343) variant due to its effect on the activity and serum levels of the ACE1 enzyme [20]. There is evidence that functional ACE1/ACE2 gene polymorphisms can generate ACE1/ACE2 imbalances, which can exacerbate lung damage in acute respiratory distress syndrome (ARDS) and may contribute to COVID-19 infection severity [21]. In this context, the study objectives were to investigate the association between ACE1 rs4343, rs4342, and rs4341 and ACE2 rs2285666 polymorphisms and the severity of COVID-19 infection among Palestinian patients using amplicon-based next-generation sequencing.

## Materials and methods

Study participants

A total of 130 frozen blood samples (5 mL each) were kept at -20°C and collected at random from the in-house blood bank in the Molecular Biology Research Laboratory, at the Faculty of Medicine, Al-Quds University, Jerusalem, Palestine. These samples were obtained from COVID-19-infected Palestinian patients and from healthy individuals in different districts in Palestine between April and May 2021. The samples were divided into three groups: control group (n=50), those who had no clinical evidence of infection and tested negative by reverse transcriptase polymerase chain reaction (PCR) (RT-PCR); case group (n=50), those who had COVID-19-positive RT-PCR and clinically diagnosed with COVID-19, regardless of the severity of symptoms; and intensive care unit (ICU) group (n=30), those who were hospitalized in the ICU due to severe life-threatening COVID-19 infection in Ramallah Hospital. All individuals aged less than 18 years were excluded because of a low expression of ACE2 in younger children and thus reduced infection [22]. Demographic information of participants such as sex, age, address, and clinical data, such as symptoms and comorbidities (smoking, diabetes, hypertension, coronary artery disease (CAD)), were collected via a questionnaire as described in our previous work [23]. The consent form was obtained from the participants at the time of blood sampling. Ethics approval was received from Al-Quds University's ethics committee under the reference number of (184/REC/2021).

Genomic DNA extraction

Genomic DNA was extracted from whole blood using NucleoSpin Blood, a mini kit for DNA from blood (MACHEREY-NAGEL GmbH & Co., Dueren, Germany), as described by the manufacturers. The concentration of DNA samples was measured by a nanodrop spectrophotometer 1000 (ND-1000) (Thermo Fisher Scientific, Inc., Waltham, MA), with concentrations ranging from 100 to 300 ng/µL. All DNA samples were stored at -20˚C until required for further analysis.

Genotyping

For genotyping, four primers (two forward and two reverse) were designed in this study using the free Primer3 v.4 online program (https://bioinfo.ut.ee/primer3-0.4.0/). Initially, each PCR was performed using two primers to amplify the target regions of ACE1 and ACE2 genes. The PCR assay was carried out in total 25 uL reaction, using 2 μL of DNA sample, 1 μL of forward and reverse primers (10 pmol each), 12.5 μL of PCR master mix (PCRBIO HS Taq Mix Red), and 8.5 μL of double distilled water. The PCR amplification condition was as follows: initial denaturation of DNA at 95°C for five minutes, followed by 35 cycles of denaturation at 95°C for 30 seconds, annealing at 63°C for 30 seconds, and extension at 72°C for 30 seconds and final extension at 72°C for six minutes. After confirming successful amplification, the four primers were then used in a multiplex PCR to simultaneously target the four SNPs (rs4343, rs4342, and rs4341 of ACE1 and rs2285666 of ACE2) in a single PCR tube.

Each forward primer was attached to the Illumina overhang adapter sequences at the 5’ end (5’-CGTCGGCAGCGTCAGATGTGTATAAGAGACA-3’), and each reverse primer was modified by adding the Illumina overhang adapter sequences at the 5’ end of it (5’-GTCTCGTGGGCTCGGAGATGTGTATAAGAGA-3) (Table 1). The reaction was carried out using 3 µL of the extracted DNA (100-300 ng/µL) in a final volume of 25 µL, which contained 12.5 µL PCRBIO HS Taq Mix Red (PCR Biosystems, Ltd., Wayne, PA), 7.5 µL double distilled water (dH2O), and 0.5 µL of each primer (10 uM). The amplification conditions were as follows: initial denaturation at 95°C for five minutes; then by 30 cycles of 95°C for 15 seconds, 60°C for 15 seconds, and 72°C for 40 seconds; and finally with a final extension step of 72°C for six minutes. The product (5 uL) was loaded on 2% agarose gel producing, two bands of DNA with a molecular size of 277 bp for ACE1 and 294 bp for ACE2. The PCR products 20 μL were cleaned by AMPure XP beads, Beckman Coulter (X1) and eluted in 25 μL elution buffer according to company protocol (https://www.protocols.io/view/magnetic-particle-based-dna-purification-e6nvwb27vmkj/v2).

**Table 1:** Primer names, sequences, and PCR products designed and used for multiplex PCR in the present study.

Gene/SNP	Primer name	Primer sequence (5′-3′)	PCR product size
ACE1/rs4343 ACE1/rs4342 ACE1/rs4341	F2_rs4343_NGS	TCGTCGGCAGCGTCAGATGTGTATAAGAGACA GCGAGCCAGCTCTGAAATTCT	277 bp
	R_rs4343_NGS	GTCTCGTGGGCTCGGAGATGTGTATAAGAGAC AGTTGATGAGTTCCACGTATTTCG	
ACE2/rs2285666	F_G8790A _NGS	TCGTCGGCAGCGTCAGATGTGTATAAGAGACA GTGAAACACACATATCTGCAATCA	294 bp
	R_G8790A _NGS	GTCTCGTGGGCTCGGAGATGTGTATAAGAGAC AGTCTTCAGCAAAATTTCCATTGTT	

DNA library preparation and bioinformatic analysis

For DNA library preparation and barcoding of each sample, Nextera XT Index Kit (Illumina, San Diego, CA) was used. Samples were deep-sequenced with the Nextseq500 machine using the 150-cycle mid-output kit (Illumina, San Diego, CA) using Macrogen Europe Company service (Amsterdam Meibergdreef, Amsterdam, The Netherlands) from the forward read direction. The obtained DNA sequences were then analyzed using the Galaxy program (https://usegalaxy.eu/). To identify the targeted polymorphisms, eight virtual probe sequences were used (six for ACE1 gene variants (three SNPs), and two for ACE2 gene variants (Table 2). All SNPs in each sample were genotyped based on the ratio between read counts for wild-type and mutant alleles.

**Table 2:** Virtual probe sequences used for genotype analysis. The targeted SNPs are underscored.

Targeted SNP	Probe sequence	Probe name	Gene
G	TGATGGCCACGT	rs4343G	ACE1
A	TGATGGCCACAT	rs4343A	
A	CAAACCCCTACC	rs4342A	
C	CAACCCCCTACC	rs4342C	
G	GGCTGGAGCTCAAGGC	rs4341G	
A	GGCTGGAGCTCAAGAC	rs4341A	
G	CTAAAAATTAGTAGCC	rsG8790	ACE2
A	CTAAAAATTAGTAGCT	rsA8790	

Statistical analysis

Analyses of categorical and quantitative values were presented as mean ± standard deviation. For comparing categorical parameters between groups, the chi-square test was used. The odds ratio (OR) and 95% confidence interval (CI) of multiple logistic regression analyses were calculated to estimate the association between genotypes and allele frequencies with the possibility and severity of COVID-19 disease and with the signs and symptoms of COVID-19 disease. Any P-value (P) less than 0.05 was considered statistically significant. IBM SPSS Statistics for Windows (version 25.0; Armonk, NY) was used for statistical analysis.

## Results

Study participants

The characteristics and comorbidities of the study groups (i.e., prevalence of smoking, diabetes, hypertension, and coronary artery disease (CAD) are shown in Table 3. Sex, prevalence of diabetes, and smoking showed no significant difference between controls versus COVID-19 patients (P=0.091, 0.177, and 0.205, respectively). However, the severity of the COVID-19 disease was associated with hypertension, CAD, and age, they were significantly higher in the ICU group (P=0.000). A significant difference in age existed among the ICU patients as they are the oldest.

**Table 3:** Demographic properties and comorbidities of the studied groups. ICU, Intensive care unit; CAD, Coronary artery disease

Variables	Case (n=50)	Control (n=50)	ICU (n=30)	Total (n=130)	P-value
Sex, n (%)					0.091
Female	31 (23.8)	22 (16.9)	12 (9.2)	65 (50.0)	
Male	19 (14.6)	28 (21.5)	18 (13.8)	65 (50.0)	
Age	36.06±19.9	32.14±13.6	56.33±17.0	39.23±19.4	<0.001
Smoking, n (%)	6 (4.6)	13 (10.0)	6 (4.6)	25 (19.2)	0.205
Diabetes, n (%)	4 (3.1)	4 (3.1)	6 (4.6)	14 (10.8)	0.177
Hypertension, n (%)	3 (2.3)	2 (1.5)	12 (9.2)	17 (13.1)	<0.001
CAD, n(%)	3 (2.3)	2 (1.5)	10 (7.7)	15 (11.5)	<0.001

The clinical characteristics of all COVID-19 patients (the cases and ICU groups) with signs and symptoms frequencies are illustrated in Table 4. The frequency of fatigue, headaches, runny noses, and loss of smell were significantly higher in the positive cases group (P=0.000) compared to the ICU group. However, dyspnea was more frequent in the ICU group (P=0.000).

**Table 4:** Clinical signs and symptoms of cases and ICU patients. ICU, Intensive care unit; P-value was obtained by the χ2 test

Variables	Case (n=50)	ICU (n=30)	Total (n=80)	P-value
Runny Nose, n (%)	28 (35.0)	7 (8.8)	35 (43.8)	0.004
Fatigue, n (%)	37 (46.3)	10 (12.5)	47 (58.8)	<0.001
Headache, n (%)	36 (45.0)	9 (11.3)	45 (56.3)	<0.001
Fever, n (%)	31 (38.8)	19 (23.8)	50 (62.5)	0.905
Loss of smell, n (%)	33 (41.3)	10 (12.5)	43 (53.8)	0.005
Muscle/body aches, n (%)	31(38.8)	20(25.0)	51(63.7)	0.674
Diarrhea, n (%)	18 (22.5)	5 (6.3)	23 (28.7)	0.064
Sore throat, n (%)	23 (28.7)	8 (10.0)	31(38.8)	0.086
Cough, n (%)	24 (30.0)	20 (25.0)	44 (55.0)	0.104
Dyspnea, n (%)	18 (22.5)	26 (32.5)	44 (55.0)	<0.001

Genotype distribution of ACE1 (rs4343, rs4342, rs4341) and ACE2 (rs2285666) polymorphisms

**Figure 1:**
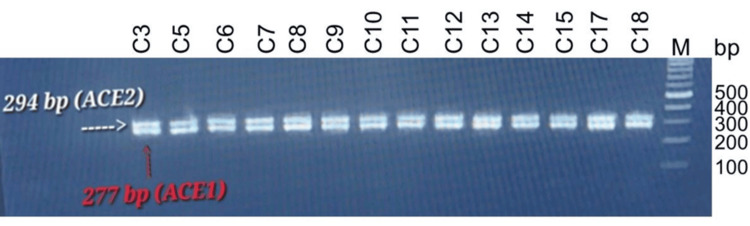
The PCR products of ACE1 and ACE2 gene fragments analyzed by agarose gel electrophoresis. Lanes C3, C5-C15, C17, and C18 are representative samples of used subjects. Lane M refers to the DNA ladder (100 bp), and the size of the upper and lower bands was approximately 300 bp (277 and 294 bp) for PCR products.

The multiplex PCR product showed two bands of DNA with a molecular size of 277 bp for ACE1 and 294 bp for ACE2, as shown in Figure 1.

The frequency and genotype distribution of ACE1 (rs4343, rs4342, rs4341) and ACE2 (rs2285666) among the three study groups are provided in Table 5. The ACE2 rs2285666 genotype distribution was marginally different between the study groups (P=0.049), while no significant differences were observed in the distribution of ACE1 rs4343, rs4342, and rs4341 genotypes (P>0.05). The ACE2 rs2285666 GG genotype was more prevalent in the COVID-19 case group compared to the control and ICU group genotypes (P=0.049) (Table 5).

**Table 5:** Genotype distribution of ACE1 and ACE2 polymorphisms in COVID-19 patients case group, ICU group, and healthy control group. P value was obtained by χ2.

Gene/SNPs	Genotype	Case (n)%	Control (n)%	ICU (n)%	P-value
ACE1/rs4343	GG	24 (18.5)	17 (13.1)	15 (11.5)	0.128
	GA	23 (17.7)	27 (20.8)	9 (6.9)	
	AA	3 (2.3)	6 (4.6)	6 (4.6)	
ACE1/rs4342	AA	34 (26.2)	26 (20.0)	18 (13.8)	0.175
	CA	14 (10.8)	23 (17.7)	9 (6.9)	
	CC	2 (1.5)	1 (0.8)	3 (2.3)	
ACE1/rs4341	GG	34 (26.2)	27 (20.8)	18 (13.8)	0.234
	GC	14 (10.8)	22 (16.9)	9 (6.9)	
	CC	2 (1.5)	1 (0.8)	3 (2.3)	
ACE2/rs2285666	GG	32 (24.6)	20 (15.4)	19 (14.6)	0.049
	GA	13 (10.0)	24 (18.5)	6 (4.6)	
	AA	5 (3.8)	6 (4.6)	5 (3.8)	

Association of ACE1 and ACE2 polymorphisms with COVID-19 infection

Logistic regression analysis adjusted for age, gender, hypertension, and CAD was used to investigate the effect of ACE1 rs4343, rs4342, and rs4341 and ACE2 rs2285666 polymorphisms in the susceptibility of COVID-19 infection, the positive case group was compared to the control group, and the ICU group was compared to the control group. GA genotype carriers of ACE2 rs2285666 among the positive case group and ICU patients were less prone to COVID-19 infection (AOR=0.208 (95% CI: 0.07-0.5), P=0.002; and AOR=0.153 (95% CI: 0.03-0.6), P=0.013, respectively). These correlations were not observed in patients with AA genotype among cases or ICU groups. In addition, no significant relationship between the ACE1 rs4343, rs4342, and rs4341 polymorphisms and COVID-19 illness was observed (Table 6).

**Table 6:** Association of ACE1 and ACE2 polymorphisms with COVID-19 infection. Adjusted-odds ratio (AOR), 95% confidence interval (CI), and P value were obtained by multiple logistic regression; Ref., Reference (Wildtype genotype).

Gene/SNP	Cases Vs. Controls AOR (95% CI)	P-value	ICU group Vs. Control AOR (95% CI)	P-value
ACE1/rs4343				
GG	Ref.		Ref.	
GA	0.55 (0.23-1.3)	0.182	0.648 (0.1-2.2)	0.486
AA	0.29 (0.06-1.4)	0.12	1.47 (0.29-7.3)	0.636
ACE1/rs4342				
AA	Ref.		Ref.	
CA	0.362 (0.02-4.5)	0.43	0.118 (0.009-1)	0.11
CC	0.779 (0.06-9.3)	0.844	0.120 (0.009-1.6)	0.111
ACE1/rs4341				
GG	Ref.		Ref.	
GC	0.500 (0.21-1.1)	0.115	1.063 (0.3-3.4)	0.918
CC	1.324 (0.11-15.8)	0.825	8.624 (0.6-116.9)	0.105
ACE2/rs2285666				
GG	Ref.		Ref.	
GA	0.208 (0.07-0.5)	0.002	0.153 (0.03-0.6)	0.013
AA	0.518 (0.12-2.1)	0.358	0.234 (0.04-1.3)	0.103

Association of ACE1 and ACE2 polymorphisms with COVID-19 signs and symptoms

Logistic regression analysis adjusted for age, sex, hypertension, and CAD showed that patients who have GA genotypes of ACE2 rs2285666 were unlikely to develop fatigue (AOR=0.405 (95% CI: 0.168-0.973); P=0.043), headache (AOR=0.277 (95% CI: 0.110-0.699); P=0.007), loss of smell (AOR=0.373 (95% CI: 0.155-0.901); P=0.028), and dyspnea (AOR=0.197 (95% CI: 0.063-0.613); P=0.005). In addition, patients who have AA genotypes of ACE2 rs2285666 were unlikely to develop dyspnea (AOR=0.843 (95% CI: 0.183-0.040); P=.029). Moreover, patients who have an AA genotype for the ACE1 rs4343 polymorphism were unlikely to develop headaches (AOR=0.188 (95% CI: 0.036-0.982); P=.048). No significant association was found for ACE1 rs4343, rs4342, and rs4341 with COVID-19 signs and symptoms (Table 7).

**Table 7:** Association of ACE1 and ACE2 polymorphisms with COVID-19 signs and symptoms. Adjusted-odds ratio (AOR), 95% confidence interval (CI), and P value were obtained by multiple logistic regression; Ref., Reference (Wildtype genotype).

Signs/Symptoms	Gene/SNP	Alleles	P-value	AOR	CI
Runny nose	ACE1/rs4343	GG	Ref.	Ref.	Ref.
		GA	0.932	0.965	0.423-2.200
		AA	0.108	0.173	0.021-1.465
	ACE1/rs4342	AA	Ref.	Ref.	Ref.
		CA	0.239	0.593	0.249-1.414
		CC	0.462	0.431	0.046-4.070
	ACE1/rs4341	GG	Ref.	Ref.	Ref.
		CG	0.281	0.62	0.260-1.479
		CC	0.472	0.438	0.046-4.145
	ACE2/rs2285666	GG	Ref.	Ref.	Ref.
		GA	0.055	0.382	0.143-1.021
		AA	0.523	1.5	0.428-5.302
Fatigue	ACE1/rs4343	GG	Ref.	Ref.	Ref.
		GA	0.527	0.777	0.356-1.698
		AA	0.092	0.291	0.069-1.224
	ACE1/rs4342	AA	Ref.	Ref.	Ref.
		CA	0.114	0.523	0.234-1.169
		CC	0.534	0.565	0.093-3.420
	ACE1/rs4341	GG	Ref.	Ref.	Ref.
		CG	0.146	0.55	0.245-1.231
		CC	0.55	0.578	0.095-3.495
	ACE2/rs2285666	GG	Ref.	Ref.	Ref.
		GA	0.043	0.405	0.168-0.973
		AA	0.969	1.02	0.309-3.387
Headache	ACE1/rs4343	GG	Ref.	Ref.	Ref.
		GA	0.188	0.58	0.257-1.306
		AA	0.048	0.188	0.036-0.982
	ACE1/rs4342	AA	Ref.	Ref.	Ref.
		CA	0.116	0.516	0.226-1.177
		CC	0.225	0.241	0.024-2.398
	ACE1/rs4341	GG	Ref.	Ref.	Ref.
		CG	0.144	0.54	0.236-1.234
		CC	0.232	0.246	0.025-2.447
	ACE2/rs2285666	GG	Ref.	Ref.	Ref.
		GA	0.007	0.277	0.110-0.699
		AA	0.207	0.395	0.094-1.670
Dyspnea	ACE1/rs4343	GG	Ref.	Ref.	Ref.
		GA	0.478	0.727	0.301-1.755
		AA	0.526	0.641	0.163-2.529
	ACE1/rs4342	AA	Ref.	Ref.	Ref.
		CA	0.39	0.681	0.284-1.635
		CC	0.839	0.819	0.120-5.608
	ACE1/rs4341	GG	Ref.	Ref.	Ref.
		CG	0.452	0.714	0.297-1.717
		CC	0.854	0.835	0.122-5.718
	ACE2/rs2285666	GG	Ref.	Ref.	Ref.
		GA	0.005	0.197	0.063-0.613
		AA	0.029	0.183	0.040-0.843
Loss of smell	ACE1/rs4343	GG	Ref.	Ref.	Ref.
		GA	0.552	1.26	0.579-2.775
		AA	0.626	0.722	0.195-2.672
	ACE1/rs4342	AA	Ref.	Ref.	Ref.
		CA	0.698	1.16	0.537-2.531
		CC	0.933	0.925	0.153-5.594
	ACE1/rs4341	GG	Ref.	Ref.	Ref.
		CG	0.605	1.22	0.565-2.667
		CC	0.95	0.944	0.156-5.703
	ACE2/rs2285666	GG	Ref.	Ref.	Ref.
		GA	0.028	0.373	0.155-0.901
		AA	0.126	0.341	0.086-1.351

## Discussion

Genetic polymorphisms of ACE1 and ACE2 have been associated with the severity rate of COVID-19. It is reported that the ACE2 rs2285666 polymorphism may influence the susceptibility to SARS-CoV-2 infection by increasing the expression of the ACE2 receptor and protein levels, by altering mRNA splicing [24]. In this study, we used amplicon based-NGS to investigate the association of ACE1 rs4343, rs4342, and rs4341 and ACE2 rs2285666 polymorphisms with severity of COVID-19 infection among Palestinian patients. This approach enabled us to successfully identify four SNPs within a single tube, offering significant advantages for future research targeting these SNPs.

In this study, the distribution of ACE2: rs2285666 genotypes had a significant difference among the studied groups (P=0.049). We noticed that the wildtype GG genotype was more prevalent in the positive case group (24.6%) compared to the control group. Such differences were not observed in any of the ACE1 rs4343, rs4342, and rs4341 variants. In comparison to another study that investigated the association of ACE1 rs4343 and ACE2 rs2285666 with COVID-19 susceptibility, it supports that COVID-19 infection risk was significantly increased in individuals carrying the GG genotype of ACE2 rs2285666 and ACE1 rs4343 [18]. This can be partially explained by the presence of other haplotypic variants among different ethnic groups, which may exert more regulatory effects on ACE2 expression.

Logistic regression analysis adjusted for possible confounding factors such as age, gender, CAD, and hypertension revealed that the GA genotype carriers of ACE2 rs2285666 among positive case group and ICU patients were less prone to COVID-19 infection (OR: 0.208 (95% CI: 0.07-0.5), P=0.002; and OR: 0.153 (95% CI: 0.03-0.6), P=0.013, respectively), indicating a protective effect of the minor allele. However, the homozygous AA genotype was not associated with COVID-19 disease, which could be attributed to the small sample size. A previous report found that age and comorbidities associated with ACE2 polymorphisms such as hypertension and CAD could exacerbate COVID-19-induced-ACE2 deficiency and increase its severity and mortality [25]. Based on the polymorphisms of ACE1 I/D and ACE2 rs2285666, researchers in a previous study in northern Spain determined that severe COVID-19 cases were associated with hypertension and high cholesterol, but the effect varies with hypertension severity [26]. This supports the current study, which revealed an association of hypertension and CAD with the severity of the COVID-19 disease as the prevalence of hypertension and CAD was significantly higher in the ICU group (P<0.05). Moreover, our study revealed that age was an independent risk factor for the severity of COVID-19 and the ICU group was older than the positive case and control groups. Similarly, a previous study has shown that older age affects the severity and prevalence of COVID-19 [18], which is apparently associated with the deterioration of the immune system with age, and comorbidities such as diabetes and hypertension further affect the immune system, increasing the risk of having COVID-19 infection. In this study, we noted that gender had no significant impact on the severity or prevalence of COVID-19 disease, contrary to previous studies finding that men are more prone to develop severe COVID-19 cases, suggesting a role for estrogen in the suppression of ACE2 expression [26].

Results of the present study regarding dyspnea, fatigue, headache, runny nose, and loss of smell were significantly higher in the positive cases group (P<0.05). A cross-sectional study was conducted on early 60,000 COVID-19 patients and reported that fever and breathing difficulties increased the likelihood of hospitalization and death. A running nose, sore throat, diarrhea, and headache, on the other hand, were associated with a lower risk of hospitalization and death; the researchers concluded that these symptoms provide protection against hospitalization and death [27]. In this study, dyspnea was more frequent in the ICU group (P<0.05), whereas the frequency of fatigue, headaches, runny noses, and loss of smell were significantly higher in the positive cases group (P<0.05). On the other hand, our results showed that ACE2 rs2285666 GA genotype carriers were unlikely to develop fatigue, headache, loss of smell, and dyspnea, indicating the association of the minor allele A with less severity of COVID-19. In addition, we found that the homozygous rs2285666 AA genotype patients were unlikely to develop dyspnea (P=0.029). For the ACE1 rs4343 polymorphism, patients who have an AA genotype were unlikely to develop headaches (P=0.048).

Our findings suggest that the ACE2 rs2285666 A-allele has a protective effect: If A-allele carriers produce more ACE2 than those with GG genotypes, they may be at least partially protected by an imbalance between ACE1 and ACE2. However, further studies with larger sample sizes are required to confirm our results. It is also possible to deal better with the adverse effects of high levels of Ang II, which can cause severe lung and heart damage [28]. It is believed that ACE2 and ACE1 have different physiological functions. Because the RAS system plays a crucial role in cardiovascular, respiratory, and diabetes pathogenesis, cross-models of ACE1 and ACE2 genotypes may aggravate COVID-19 by causing RAS imbalance by increasing the ratio of ACE1-to-ACE2. Acute respiratory distress syndrome (ARDS) is associated with the imbalance of ACE2 activity in favor of ACE1 activity, leading to a RAS imbalance and greater lung damage. This may be due to the reduction of pulmonary angiotensin (ANG 1-7) levels and the elimination of its anti-inflammatory effects [5].

A study conducted on 2,504 subjects from five populations calculated genetic expression risk scores for ACE2 variants using 1,000 Genomes Project data. It was reported that South Asians and East Asians showed the highest ACE2 expression, while Africans had the lowest. These findings suggest that variations in gene expression may influence COVID-19 transmission, severity, and prognosis across different populations [29]. Khayat et al. found 10 ACE2-related variants in coding, noncoding, and regulatory sites, which can explain the epidemiological differences associated with COVID-19 [15].

Our study has several limitations. Firstly, the sample size was relatively small, which may limit the generalizability of the findings. Moreover, the lack of information on the vaccination status of the study subjects could have influenced the variability of signs and symptoms. The presence of different variants of SARS‑CoV‑2 (e.g., Alpha, Beta, Delta, and Omicron), each with different manifestations and pathogenesis, also likely contributed to the severity of signs and symptoms among the studied subjects. However, our findings provide preliminary insights for further investigations in various ethnicities. Thus, future research with a larger sample size should be conducted to replicate these findings and to investigate the role of human genetics in various signs and symptoms of COVID-19.

## Conclusions

Our study found that the genotype distribution of the ACE2 rs2285666 polymorphism showed a difference between COVID-19 patients and the control group, indicating that individuals with the GG genotype are more prevalent among COVID-19 patients. In contrast, no significant differences were observed in the genotype distributions of the ACE1 variants (rs4343, rs4342, rs4341). Additionally, age and comorbidities such as hypertension and CAD were identified as independent risk factors for COVID-19. COVID-19 severity might be predicted by the ACE2 rs2285666 SNP, helping identify susceptible population groups, in order to improve disease control.
